# Activation of Autophagy in a Rat Model of Retinal Ischemia following High Intraocular Pressure

**DOI:** 10.1371/journal.pone.0022514

**Published:** 2011-07-22

**Authors:** Antonio Piras, Daniele Gianetto, Daniele Conte, Alex Bosone, Alessandro Vercelli

**Affiliations:** 1 Neuroscience Institute of the Cavalieri Ottolenghi Foundation, Orbassano, Torino, Italy; 2 Department of Anatomy, Pharmacology and Forensic Medicine, Neuroscience Institute of Turin, University of Turin, Turin, Italy; 3 Italy Department of Oncological Sciences, Institute for Cancer Research and Treatment (IRCC), University of Turin, Candiolo, Turin, Italy; Federal University of Rio de Janeiro, Brazil

## Abstract

Acute primary open angle glaucoma is an optic neuropathy characterized by the elevation of intraocular pressure, which causes retinal ischemia and neuronal death. Rat ischemia/reperfusion enhances endocytosis of both horseradish peroxidase (HRP) or fluorescent dextran into ganglion cell layer (GCL) neurons 24 h after the insult. We investigated the activation of autophagy in GCL-neurons following ischemia/reperfusion, using acid phosphatase (AP) histochemistry and immunofluorescence against LC3 and LAMP1. Retinal I/R lead to the appearance of AP-positive granules and LAMP1-positive vesicles 12 and 24 h after the insult, and LC3 labelling at 24 h, and induced a consistent retinal neuron death. At 48 h the retina was negative for autophagic markers. In addition, Western Blot analysis revealed an increase of LC3 levels after damage: the increase in the conjugated, LC3-II isoform is suggestive of autophagic activity. Inhibition of autophagy by 3-methyladenine partially prevented death of neurons and reduces apoptotic markers, 24 h post-lesion. The number of neurons in the GCL decreased significantly following I/R (I/R 12.21±1.13 vs controls 19.23±1.12 cells/500 µm); this decrease was partially prevented by 3-methyladenine (17.08±1.42 cells/500 µm), which potently inhibits maturation of autophagosomes. Treatment also prevented the increase in glial fibrillary acid protein immunoreactivity elicited by I/R. Therefore, targeting autophagy could represent a novel and promising treatment for glaucoma and retinal ischemia.

## Introduction

Retinal ischemia, a common cause of blindness worldwide, involves reduced blood flow and impaired diffusion of oxygen; it is associated with acute and chronic glaucoma (primary open angle glaucoma, POAG), central or branch retinal arterial occlusion (CRAO/BRAO), retinal detachment, and diabetic retinopathy [1], [2], [3], [4]. In rats, ischemia associated with high intraocular pressure (IOP) [5], [6] produces pathological features that are almost identical to those reported for CRAO and POAG in humans.

Ischemia/reperfusion (I/R) injury is characterized by retinal degeneration, including extensive loss of neurons in the ganglion cell layer (GCL) and in the inner nuclear layer (INL) [7] the extent of the insult and the severity of neuronal death are related to the duration of ischemia, or the degree of IOP elevation [8], [9], [10]. Three modes of cell death, apoptosis, necrosis and autophagic death, have been described [11], [12], [13]: the first two, which can be readily identified, have been extensively investigated, whereas autophagic death has only recently garnered attention as a significant contributor to ischemia associated damage.

Three main forms of autophagy, chaperone-mediated, micro- and macroautophagy (hereafter simply called autophagy) have been described [14]. In eukaryotes, autophagy is a physiological process that leads to the degradation of long-lived proteins, cytoplasmic organelles and toxic agents by degradation in pre-existing lysosomes [15]. Lysosomes, which contain different acid hydrolases, fuse with the new autophagic vacuole and load degradative enzymes into it [16]. Autophagy-associated cell death is caspase-independent, necrosis like [17], [11], [18] and apparently operates as an alternative mechanism when apoptosis has been compromised [19]. However, recent findings demonstrate the strong correlation with caspases [20]. Autophagic death is detected during development and tissue remodelling [21], [22], subsequent to ischemia/hypoxia [23], [24] and in a number of neurodegenerative diseases [25] in the retina, autophagy has been observed during development [26], in response to light exposure [27].

In this paper we document the occurrence of autophagic retinal cell death following I/R produced by acute IOP increase. We showed earlier that this model of I/R induces apoptotic cell death [10]; we now extend these observations to demonstrate that I/R also induces (i) autophagic activity, (ii) the formation of lysosomal vacuoles (detected by their content of acid phosphatase, AP), and promotes (iii) enhanced endocytosis, a process characteristic of dying neurons [28], [29], [30].

Taken together these results demonstrate enhanced autophagic flux. Moreover, our studies underline the important relationship between autophagy and apoptosis in the control of cell death after I/R, bearing implications for the development of potential neuroprotective therapies that are aimed at preventing ischemia-related cell death.

## Results

### Acid phosphatase histochemistry

**Figure 1:**
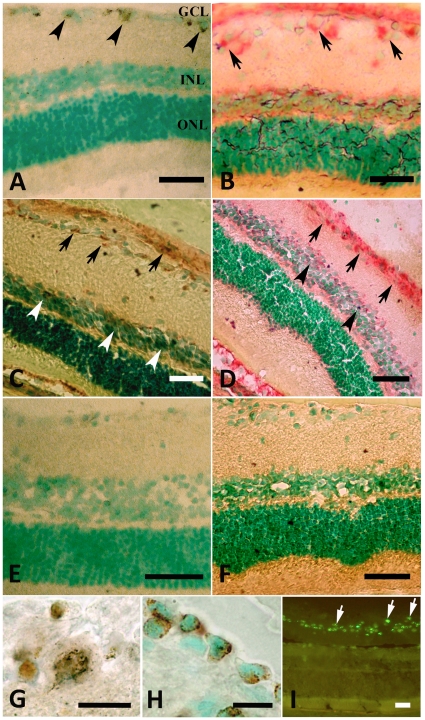
AP and endocytic activities in the retina. A–F: acid phosphatase (AP) activity is visualized with Gomori's (A, C, E) and Barka & Anderson (B and D) staining, counterstained with methyl green. 12 h after I/R, positive cells are located only in the ganglion cell layer (GCL), and are identified by their content of typical brown cytoplasmic granules (arrowheads in A) and by the red reaction (B, arrows) in the GCL and INL. 24 h after I/R, AP-positive cells are visible in both the GCL (arrows) and INL (arrowheads) (C–D). Only weak staining in the GCL is seen with the Gomori technique 48 h after I/R (E). No labeling is found in control retinas (F). Markedly positive cytoplasmic granules are visible in GCL-cells at higher magnification (G). H–I: 24h after I/R and intravitreal injection of HRP (H) or 4.4 kDa FITC-labelled dextran (I, arrows), corresponding granules are visible in neurons. GCL  =  ganglion cell layer; IPL  =  inner plexiform layer; INL  =  inner nuclear layer; OPL  =  outer plexiform layer; ONL  =  outer nuclear layer. Scale bars  =  100 µm and 10 µm (G and H).

Overall, retinal morphology was conserved following I/R (Figure 1). In I/R retinas, AP activity was detected at 12 h following I/R (Figure 1 A–B), was maximal by 24 h (Figure 1 C–D) and declined at 48 h (Figure 1 E). Both methods used to visualize enzyme activity showed robust staining at 24 h post-insult, although staining was darker with the Barka and Anderson technique. Most intense staining was localized to GCL; sporadic positively stained cells were also visible in the inner nuclear layer (Figure 1 C–D, arrowheads). At high magnification, Gomori staining revealed clusters of small, intensely stained granules, preferentially located in the periphery of the cytoplasm (Figure 1 G), as is characteristic of lysosomal systems. Almost all GCL-neurons were stained, but to different degrees: the larger the cell the more intensely reactive it was. The use of NaF in the incubation medium resulted in a complete inhibition of enzymatic activity. In control sections non-specific reactivity for AP was detectable in retinal neurons (Figure 1 F).

### Labelling endocytosis *in vivo*

In response to I/R, endocytotic activity in retinal neurons was reflected in a strong, selective uptake of horseradish peroxidase (HRP) or of fluorescein isothiocynate (FITC)-labelled dextran. Twenty-four hours following I/R, HRP- or FITC dextran-positive granules were visible in ganglion cell layer, (Figure 1 H and I, respectively): labelling extended throughout the cells, and was particularly robust following HRP uptake.

### Lysosomal and autophagosomal activity

**Figure 2:**
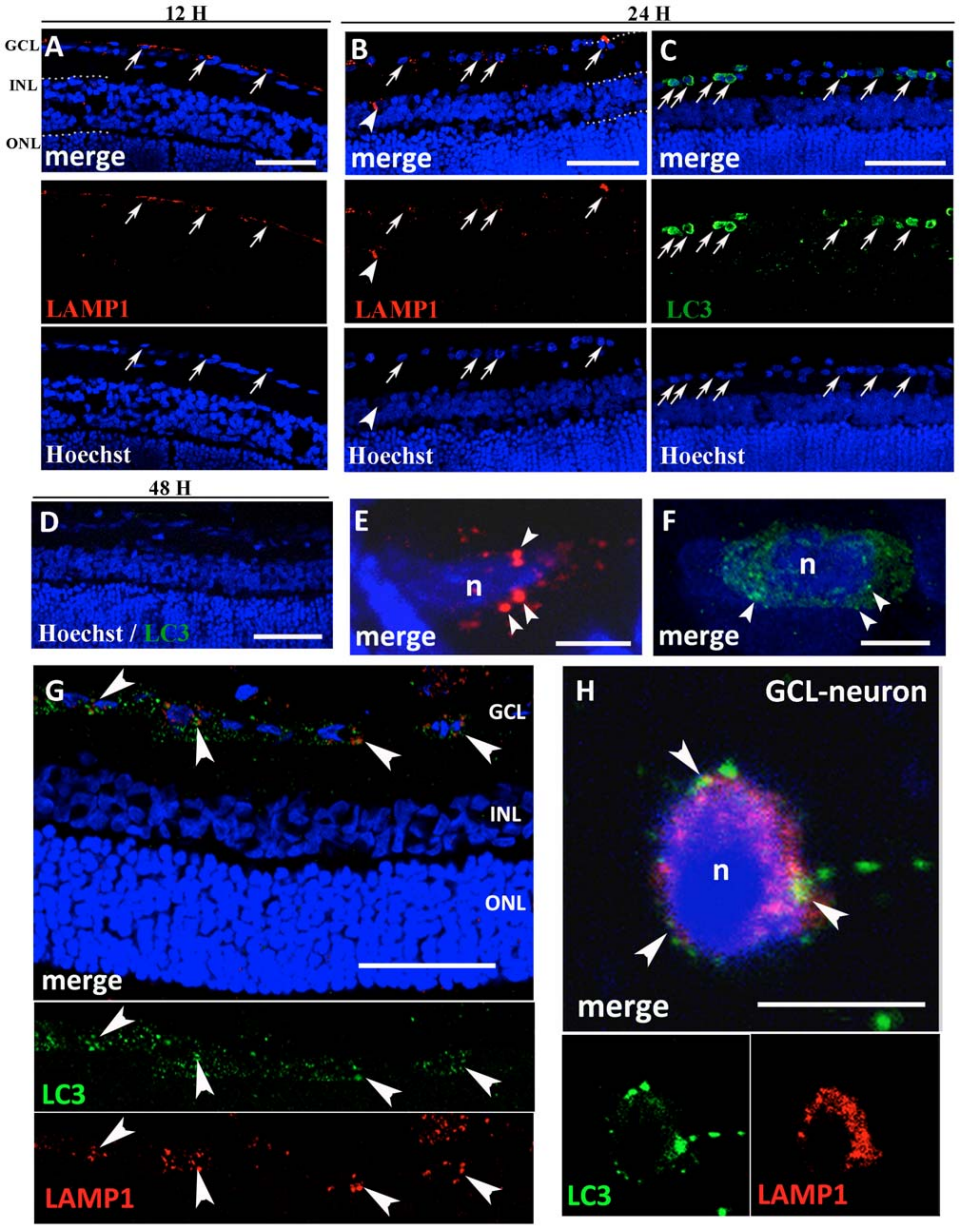
Immunofluorescence against LAMP1 and LC3. All sections were counterstained with bisbenzimide (Hoechst staining) to show retinal layers. Twelve hours after I/R, LAMP1 positive cytoplasmic granules are present (A) and 24 h after I/R both LAMP1 (B) and LC3 (C) positive granules are present in the GCL (arrow) and INL (arrowheads). LC3-positive vesicles are more represented than LAMP1 vesicles. LC3-immunopositivity is absent after 48 h (D). At high magnification (E–F), clear cytoplasmic lysosomal LAMP1 positive vesicles (arrowheads) can be observed in the GCL at 24 h from the insult (E). LC3 labelling appear as numerous fluorescent dots (arrowheads) after 24 h from the I/R (F). Double immunolabeling against LAMP1 (red) and LC3 (green) at 24 h shows the relationship between autophagosomes and lysosomes (G). The increase in punctuate LC3 and LAMP1 occurs in the same neurons after 24 h (G, arrowheads). At high magnification, confocal microscopy reveals that autophagic marker and LAMP1 colocalize, suggesting that fusion of autophagosomes with lysosomes occur in dying neurons after I/R (arrowheads, H). Abbreviations as in Figure 1. Scale bars = 50 µm (A); 100 µm (B, C, D and G) and 5 µm (E, F and H).

Twelve and twenty-four hours after the I/R, lysosome-associated membrane protein-1 (LAMP1) immunostaining was detectable in the GCL (Figure 2, arrows in A), at high magnification, it was possible to appreciate intensely labelled cytoplasmic lysosomal vesicles (Figure 2 E). Only 24 h after the insult, in the GCL, frequent cells were positive for fluorescently tagged light chain 3 (LC3) positive structures (Figure 2 C) throughout the cytosol (Figure 2 F). LC3 is the only known mammalian protein identified that stably associates with the autophagosome membranes. Thus LAMP1 and LC3 immunopositivities were both present at 24 h (Figure 2 B and C) and both disappeared after 48 h (Figure 2 D). In fact, in the early stage, the initial vesicle, i.e. the phagophore, has formed and subsequently the LC3 complex associates to the developing double autophagosome membrane, demonstrating the autophagosome formation [31]. We investigated the relationship between autophagic and lysosomal activity using double-immunolabeling for LC3 and LAMP1 at 24 hours. Most LC3-positive neurons displayed also an increase in LAMP1 labelling, underlining that the two activities occurred in the same cells (Figure 2 G). At high magnification, fusion of autophagosomes with lysosomes could be appreciated (Figure 2 H, arrowheads).

### Expression of LC3 in IOP retina after 24 h

**Figure 3:**
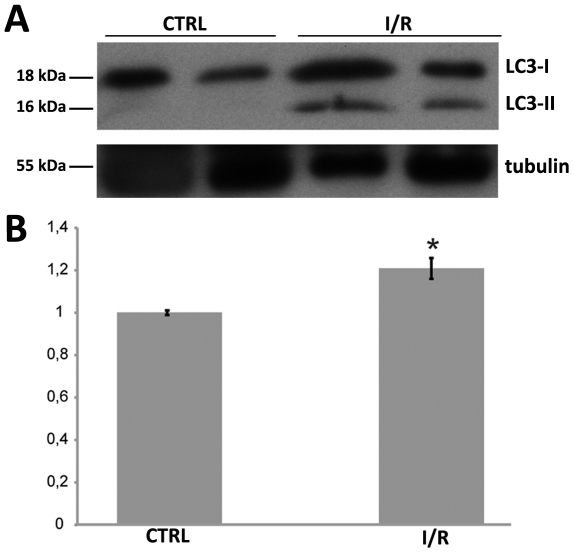
Immunoblot analysis of LC3 I and II expression in the retina after IOP. (A) Autoradiography of the western blot probed with anti-LC3 and anti-βIII tubulin antibodies. (B) Quantitation of the image in (A) after normalization with βIII tubulin. Level of LC3-II in retinas increase by 20% 24 hours after IOP compared with the control (n = 2, *P<0.05). LC3-I expression in the retina does not change significant after IOP (data not shown in B).

Autophagy induction was determined by using the expression levels of the autophagy protein LC3. To evaluate the increases in autophagy flux after IOP we probed the retinal lysates with anti-LC3 antibody. The western blot analysis demonstrates that the antibody recognizes two LC3 isoforms, 18 (LC3-I) and 16 kDa (LC3-II). In IOP retinas LC3-II was upregulated approximately 20% compared with the sham (Figure 3). The intensities of the signals of LC3-I and LC3-II were normalized with tubulin.

### Relationship between autophagic and apoptotic death

To evaluate the relationship between autophagic and apoptotic mechanisms, we investigated the expression of cleaved caspase-3, a critical executioner of apoptosis; it is partially or completely responsible for the proteolytic cleavage of many key proteins [32]. However, the association of several markers is required for appropriate detection of apoptotic cells [33]. Therefore, the identification of cleavated caspase-3 positive neurons should be related to other apoptotic markers, such as Terminal deoxynucleotidyltransferase-mediated biotinylated UTP Nick End Labeling (TUNEL)-staining.

**Figure 4:**
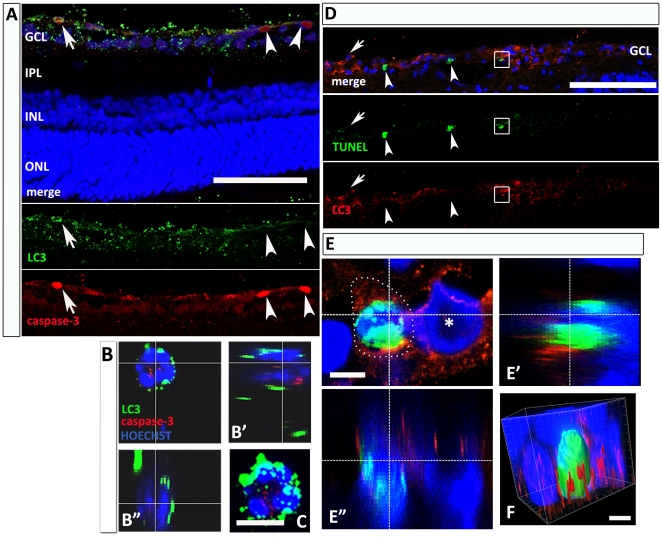
Relationship between autophagic and apoptotic neuronal death. (A) Double immunolabeling shows that cleaved caspase-3 is detectable in both LC3-positive (arrow) and negative (arrowheads) GCL-neurons, 24 h after injury. At high magnification of a GCL-neuron (B), LC3 (green) and cleaved caspase-3 (red) are colocalized in the same cell, as visible in B’ and B” rotations along the x- and y-axes. (C) Superposition of the confocal stacks. (D) Photomicrographs showing neurons labeled with TUNEL-staining (green) and immunofluorescence against LC3 (red) in the retina 24 h after I/R. The merge image (upper panel) shows a fraction of TUNEL-positive neurons having cytoplasmatic LC3-positive vesicles (box), and other neurons positive for either marker (LC3: arrow; TUNEL: arrowheads). (E) GCL-neurons double-positive at high magnification; apoptotic- and autophagic-markers were expressed in the same neuron (E’ and E” rotations along x- and y-axes). (*) GCL-neuron LC3- but not TUNEL- positive. (F) 3-D reconstruction of a double positive neuron in E, where the LC3-positive vesicles (red) surround the TUNEL-positive nucleus (green) (Imaris® Software). Abbreviations as in Figure 1. Scale bar: 100 µm (5 µm in C, E and F).

At 24 hours post-I/R, LC3 and cleaved caspase-3 double labeled neurons were detected (Figure 4, arrow in A and high magnification in B). Nevertheless, we could also find single labeled neurons for each marker, thus suggesting that autophagy and apoptosis do not necessarily overlap and may occur independently or at different time points from each other (Figure 4, arrowheads in A). I/R in retina increases both autophagic [34] and apoptotic [10], [35] cell death.

TUNEL-staining gave similar results at 24 h (Figure 4 D): it was possible to demonstrate the presence of autophagic vesicles in TUNEL-positive GCL-neurons (box). However, cells single positive for LC3 (arrow) and TUNEL (arrowheads) were also found.

### 3-Methyladenine treatment inhibits autophagy, decreases caspase-3 cleavage in GCL-neurons and prevents neuronal death following injury

**Figure 5:**
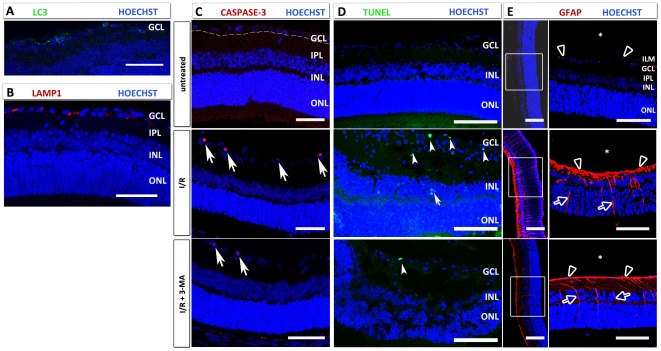
Effects of 3-MA treatment in I/R after 24 h. In 3-MA-treated I/R retinas treated, LC3-positivity is markedly reduced (A), whereas lysosomal activity (LAMP1) is unchanged (B) compared to I/R retinas. (C) Cleaved caspase-3-positive cells are absent in the untreated retinas (upper panel), whereas a strong increase in cleaved caspase 3-positivity in the GCL is seen at 24 h (middle panel), significantly prevented by 3-MA treatment (bottom panel). (D) TUNEL-positive neurons are found occasionally in the control retina, whereas they increase dramatically after I/R in the GCL (arrowheads) and in INL (arrow). This increase in TUNEL-positive cells is prevented by 3-MA treatment (bottom panel). (E) I/R increase GFAP immunoreactivity (red) in the retina: in the untreated retina, only the end feet of the Müller cells (arrowheads) are GFAP-positive. GFAP expression strongly increase 24 h after I/R, and is prevented by 3-MA administration. In the I/R and I/R + 3-MA retinas, GFAP positivity is detectable in the end feet (arrowheads) and radial processes (arrows) of Müller cells. Following 3-MA treatment, the immunoreactivity is decreased versus I/R retina especially in the end feet of Müller cells. ILM: inner limiting membrane. Abbreviations as in Figure 1. (*): vitreous. Scale bar: 50 µm (100 µm in D).

To evaluate whether 3-Methyladenine (3-MA) inhibited autophagic and lysosomal activity, we studied the LC3 and LAMP1 expression in GCL-neurons 24 h after increase in IOP and treatment with 3-MA: rare LC3-positive vacuoles were observed (Figure 5 A), while spread LAMP1 vesicles were diffused in the cytoplasm (Figure 5 B). Therefore we demonstrated that 3-MA inhibits autophagy but not lysosomal activity. Cleaved caspase-3 (Figure 5 C) and TUNEL positive (D) in the retina following I/R (middle panel) were reduced following 3-MA treatment (lower panel) compared to untreated (upper panel).

### Inhibition of autophagy prevents reactive astrogliosis in the retina

We also studied the effects of 3-MA on glial fibrillary acidic protein (GFAP) expression, the main intermediate filament specific for mature astrocytes in the central nervous system in normal and in pathological conditions [36]. In fact, in the control retina, GFAP was located exclusively in the end feet of the Müller cells (Figure 5E – top panel) creating the inner limiting membrane (ILM). Following I/R, GFAP immunoreactivity was strongly upregulated in particular in the end feet and in the radial processes of the Müller cells (Figure 5 E – middle panel).3-MA reduced the activation of Müller cells particularly visible in the inner processes and end feet (Figure 5 E – bottom panel).

### Effects of I/R +/− 3-MA treatment on the number of GCL-neurons

**Figure 6:**
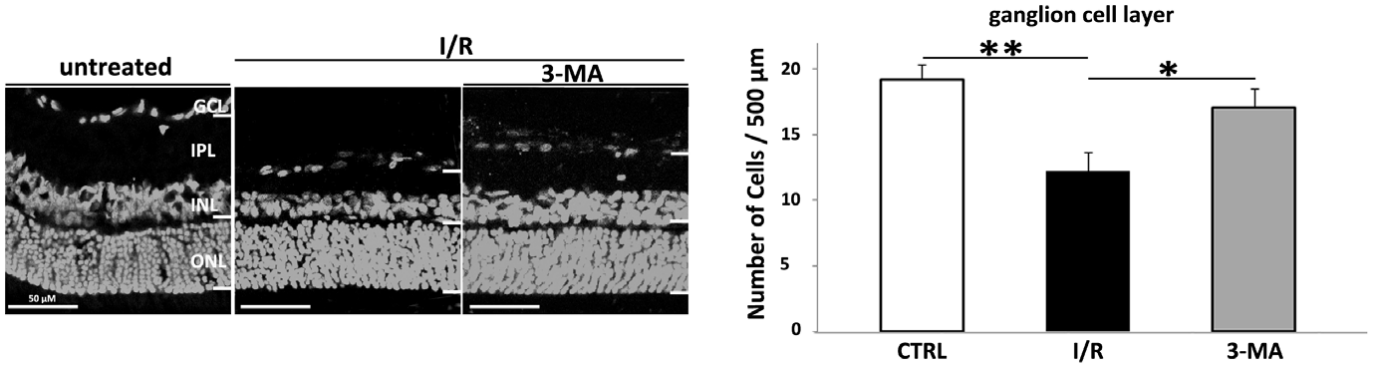
Cell counts in the GCL. Representative transverse sections through rat retina at 24 h, Hoechst staining. Retinal thickness is markedly reduced, compared to the control (A), after injury (B), mainly due to decrease in IPL thickness and number of GCL-neurons; these effects are partially prevented by 3-MA treatment (C). No statistical significant alterations are apparent in the other layers. (D) Quantification of GCL-neurons numbers in each group: in the I/R retina, the number of the GCL-neurons significantly decrease compared to controls (** *P<0.01*); 3-MA partially prevent neuron death (* *P<0.05*). Scale bar: 50 µm.

Counts of GCL-neurons following I/R showed a significant decrease in GCL-neurons number in rats treated with the vehicle alone (12.21±1.13 cells/500 µm), compared to controls (19.23±1.12 cells/500 µm); this decrease was partially prevented by 3-MA (17.08±1.42 cells/500 µm, *P<0.05) (Figure 6).

## Discussion

The present study investigated the involvement of autophagy in a rat model of ischemia/reperfusion after elevated IOP. Increased IOP leads to a significant amount of apoptosis in the rat retina [10], as indicated by the activation of caspase-3-mediated mechanisms and by the presence of TUNEL-stained neurons. In addition, retinal ischemia also causes necrotic cell death [37]. Here we show that I/R leads to the appearance of AP-positive granules, to the increase in LC3-II and LAMP1 expression and to enhanced endocytosis of both HRP and FITC labelled dextran in GCL-neurons.

In our experiments, AP-positive granules, characteristic of lysosomes, were present 12 and 24 h after the insult in GCL-neurons. An increase in lysosomal profiles has also been observed ultrastructurally in the ischemic brain under electron microscopy [23], but the molecular pathway linking I/R to autophagy is still poorly understood: NMDA induced cell death in dissociated neuronal cultures activates autophagy via a mechanism that is probably dependent on JNK [38], [39], and in the cortex hypoxia/ischemia is a potent trigger of autophagy, due to the activation of an ER resident translation initiation factor [40].

In order to exclude that the increase in LC3-II expression was caused by a reduction in lysosomal activity or that a defect in autophagosome-lysosome fusion caused vesicular retention in the cytoplasm [41], we evaluated the expression of lysosomal marker. We, showed that the expression of LAMP1, a major constituent of the lysosomal membrane, was increased in damaged GCL-cells from 12 h after I/R, before the finding of LC3 positivity (24 h), but both disappeared at 48 h: this could support the hypothesis that the marked positivity for autophagosome in the GCL-neurons reflect an increase in the autophagic activity more than an inhibition of their clearance. By double-immunofluorescence staining, we confirmed that fusion of autophagosomes with lysosomes occurred in neurons after I/R, underling that the autophagic process was in progress.

LC3-II is the only known protein that specifically associates with autophagosomes [42], whereas the protein LGP120 (LAMP1) is a lysosomal marker; during autophagosome formation LC3-I isoform is converted into LC3-II and then located at both inner and outer membrane. Our antibody cannot discriminate between LC3-I and LC3-II isoforms, but from the subcellular localization of labelling we can infer that it refers to LC3-II: in fact, LC3-I localizes in the cytosol and its immunoreactivity is diffused, whereas LC3-II is membrane-associated and its immunoreactivity is localized in autophagosomes [43].

In addition, we performed LC3 immunoblot analyses and densitometry in control and IOP retina extracts: in fact, the unconjugated (LC3-I) and conjugated (LC3-II) forms can be easily separated by SDS-page. The amount of LC3-II correlates with the number of autophagosomes and immunoblotting of endogenous LC3 represents a relevant method to measure autophagic activity [42]. We found that protein band densities of LC3-I and LC3-II levels in retinas 24 h after IOP were increased compared to control retinas. Taken together, our results indicate that the autophagic flux increases in the retina after IOP.

Clearance of autophagosomes occurs via fusion with lysosomes [44]. In our experiments, the positivity for the lysosomal markers appeared at 12 h. We also studied the relationship between endocytosis and autophagy. These processes are profoundly related to each other, even though endocytosis can occur also in other types of cell death. Clarke and coworkers have documented the occurrence of endocytosis and autophagic cell death in the isthmooptic nucleus of the chick embryo following deafferentation [28], [29] or after blockade of retrograde trophic maintenance from the retina [45]. In the cortex, increased endocytosis precedes cell death [38] and inhibitors of clathrin-mediated endocytosis block excitotoxic cell death in cultured hippocampal neurons [46]. On the other hand, Borsello et al. [30] showed that endocytosis is not a constant feature of all apoptotic neurons and can occur even in the absence of autophagic cell death.

Our findings show that the I/R caused by increased IOP enhances endocytosis of both HRP and FITC labelled dextran into GCL neurons, one day after the insult. We can exclude that the tracers enter the cells across leaky plasma membranes, since the granules are clearly concentrated in round structures of a size comparable to that of endosomes. Moreover, the large size of HRP would prevent it from entering across cell membranes. Activation of autophagy *in vivo* may represent a protective mechanism used by cells [47]: autophagy genes delay cell death, and the process of autophagy itself may represent a defense mounted by the cell against starvation [48]. Autophagy can also be activated for the purpose of cellular autolysis and self clearance [49], or as a mechanism to remove toxic, multimeric complexes that eventually promote cell death in neurodegenerative diseases [25]. In addition, in many neurodegenerative disorders altered proteins are first degraded through either the ubiquitin-proteasome system, or via chaperone-mediated autophagy, and impairment of these mechanisms promotes protein aggregation [50], [25]. Interestingly, there is a progressive deterioration in autophagic mechanisms with aging [51], [52].

On the other hand, autophagy may promote cell death through excessive self digestion, and via the degradation of essential cellular constituents [53]. Several autophagy-related proteins participate in the different steps of autophagy [54]. Autophagic markers such as Beclin-I (Bcl-2-interactin protein) and LC3 (microtubule associated protein 1 light chain 3) are increased in the penumbra of an area of cerebral focal ischemia in the two days following the insult [24]; such increase might represent both a mechanism to recycle damaged material and to lead to cell death.

To verify this controversial hypothesis, we have used a widely used pharmacological inhibitor of autophagy in mammalian cells, 3-MA. 3-MA inhibits the activity of the class III phosphatidylinositol kinase (PI3K), the mammalian homolog of yeast vps34. The latter is required for protein sorting from the Golgi to the lysosome or vacuole in yeast [55]. Therefore 3-MA potently inhibits maturation of autophagosomes and has been commonly used to understand the role of autophagy [56], [57], [58], [59], [60].

The inhibition of autophagy by 3-MA suppressed autophagosome formation and the positivity for apoptotic markers (cleavated caspase-3 and TUNEL staining), thus reducing cell death, underlining that apoptosis and autophagy pathways are intricately intertwined in the cell [20], [61], [62], [63]. By blocking the autophagic signaling pathway, 3-MA has a significant impact on the propagation of the apoptotic signals, as shown by Wang et al. (2009) [64] who demonstrated that it reduces DNA fragmentation in the striatum induced by quinolinic acid.

Moreover, the antiapoptotic protein Bcl-2 is also an antiautophagy protein via its inhibitory interaction with Beclin-I [65]. Beclin-I colocalizes with activated caspase-3 [24] suggesting that autophagy could represent an associated/alternative pathway of cell death which may become particularly relevant when caspase-mediated apoptosis is blocked [26]. Another relevant issue regarding autophagic cell death is whether it represents an independent mode of cell death or it occurs in other types of cell death, of which it may represent itself a step [26]. Our results showed that autophagy and apoptosis can coexist in the same damaged neurons after I/R. In fact in different studies, apoptosis and autophagy have been found to share common molecular aspects and they might coexist in the same cell [66]. Nevertheless, we could also find neurons reactive only for either marker, thus suggesting that autophagy and apoptosis do not necessarily overlap and may occur independently from each other. Certainly, autophagic and apoptotic pathways show common upstream signals and their functional relationship is complex [67] and deserves further studies.

Moreover it has been demonstrated that several neurodegenerative diseases elicit the reactive Müller cell gliosis with a rapid and massive GFAP accumulation [68]. Müller cell represents the main retinal glial cell that orchestrates the retinal response after damage [69]. 3-MA treatment prevented astrogliosis in damaged retinas at 24 h, compared to control, demonstrating that inhibition of autophagy partially prevents Müller cells activity during I/R injury.

In conclusion, we have shown not only an enhancement in autophagosome formation but also an increase in lysosomal activity after IOP in the retina, suggesting the improvement of autophagy flux. However, it is unclear whether the enhancement of autophagy activities was due to a defect in lysosomal fusion causing an accumulation of autophagosomes. Nevertheless, the absence of autophagic vesicles at 48 h showed that the process was in progress and they had been degraded.

There is a growing interest in the role of autophagy in neurodegenerative diseases and following ischemia, and an emerging consensus that autophagy represents a double edged sword, representing alternatively a protective and prosurvival mechanism, or part of a pathway leading to cell death. Targeting autophagy, either by inhibition or by enhancement, could represent a novel and promising tool in the treatment of diseases of the nervous system, in retinal ischemia as well as shown for Alzheimer's and Parkinson's diseases [70] and in a neonatal model of cerebral ischemia [71].

## Materials and Methods

### Ethics statement

All animal experimental procedures were approved by and carried out in accordance with the European Communities' Council Directive of 24 November 1986 (86/609/EEC), authorization number 17/2010-B of 30 June 2010 by Italian Department of Health, University of Torino's institutional guidelines on animal welfare (DL 116/92) and were approved by the University of Torino ethical committee; efforts were made to minimize suffering.

### Animals

Adult male Wistar albino rats (4–7 weeks of age) from the animal colony in the Department of Anatomy, Pharmacology and Forensic Medicine at the University of Torino were housed with a 12 h light/dark cycle, and given free access to food and water. I/R was induced in one group of rats (N = 24) (Table 1). Four animals of the first group were used for labelling endocytosis, as described below, and three animals were treated with 3-Methyladeninde (3-MA). All efforts were made to minimize the number of animals used.

**Table 1:** Experimental plan.

# Rats^1^	Procedure	Survival	Histological/Immunohistological procedures^2^
4	1 h I/R	12 h	AP histochemistry/IF LC3, LAMP1,CASPASE-3
9	1 h I/R	24 h	AP histochemistry/IF LC3, LAMP1,CASPASE-3/TUNEL assay
4	1 h I/R	48 h	AP histochemistry/IF LC3, LAMP1,CASPASE-3
2	1 h I/R	24 h	Intravitreal HRP 3 h before sacrifice, HRP histochemistry
2	1 h I/R	24 h	Intravitreal FITC-dextran 3 h before sacrifice
3	1 h I/R+3MA	24 h	IF LC3, LAMP1, CASPASE-3/TUNEL assay
2	1 h I/R	24 h	Immunoblot analysis LC3 I and II

1 For each animal, the left eye was injected, the right eye served as control.

2 IHC was performed on 2 animals of each group.

### Induction of I/R by elevated IOP

Induction of I/R was achieved according to the method of Takahashi et al. [7]: rats were anaesthetized by inhalation of 1.5%–3.5% iso fluorane (Forene™, Deutsche Abbott GmbH, Wiesbaden, Germany) vaporized in a 30/70 mixture of O_2_/N_2_O using a face mask; 0.5% proparacaine hydrochloride solution (an ophthalmic topical anaesthetic, Bausch & Lomb Inc., Tampa-FL) was applied onto the eye. Under the operating microscope (Carl Zeiss Inc., Jena, Germany) a 27-gauge needle, connected to a reservoir containing 500 ml sterile saline, was inserted into the anterior chamber of the left eye. IOP was raised to 110 mmHg by elevating the reservoir 149.6 cm above the animal's eye [72], [73]. The infusion needle was removed from the anterior chamber after one hour, and the IOP was allowed to return to normal levels. The right eye of each rat was cannulated, but was maintained at a normal IOP (28 mmHg) [74] for one hour, as a control. Animals were sacrificed at 12, 24, or 48 h after the increase in IOP.

### Acid phosphatase histochemistry

Two histochemical techniques were used for detecting AP activity in the ischemic retina: the technique described by Barka and Anderson [75] was applied for detecting lysosomal enzymes, and the Gomori method was used to study lysosomal membrane activation [76].

For the Gomori procedure, sections were thawed, allowed to dry for 1 hour at 37°C, washed three times in saline, and once in distilled H_2_O (dH_2_O) at room temperature; they were immersed in the incubation medium containing 0.68 mg/ml Pb(NO_3_)_2_ and 4.91 mg/ml sodium glycerophosphate in 0.2 M acetate buffer (pH 4.7) for 90 min at 37°C. Some sections were incubated in medium to which 0.01 M NaF (an AP inhibitor) was added; these served as a negative control (i.e., for no AP activity). The AP reaction was stopped by rinsing slides for 5 min in each of four dH_2_O washes. Endogenous AP activity was revealed by immersing sections in 1∶200 ammonium sulphate in dH_2_O for 10–20 seconds, or until a brown precipitate appeared. Sections were rinsed, coverslipped in 1∶1 phosphate buffer (PB)-glycerol, and analyzed on a Nikon Eclipse E800 light microscope.

For a modified Barka and Anderson procedure, slides were incubated for 45 minutes at 37°C in a ready-for-use solution containing naphthol AS-BI phosphate, di-methylformamide, pararosanilin in 0.2 M acetate buffer (pH 5.6), and 4% sodium nitrate (Bio-Optica, Milan, Italy); they were washed in dH_2_O for 10 minutes, counterstained with buffered methyl green (Bio-Optica) for 5 minutes, and coverslipped in 1∶1 phosphate-buffered saline (PBS)-glycerol. This method reveals enzymatic activity by a red precipitate, while nuclei are labelled green.

### Immunofluorescence

At different time intervals (12, 24 and 48 h) from I/R, rats were killed with an overdose of anaesthetics and perfused transcardially with 4% paraformaldehyde in 0.1 M PB, pH 7.4. Their eyes were dissected out, post-fixed in fixative for three hours, cryoprotected overnight in 30% sucrose in 0.1 M phosphate buffer (pH 7.2), embedded in cryostat medium (Bio-Optica, Milan, Italy) and frozen. Sections were cut on the cryostat at a thickness of 10 µm, mounted onto gelatin-coated slides, and stored at −20°C until they were reacted for immunohistochemistry. The sections were incubated overnight at 4°C with the following antibodies:

i) rabbit polyclonal anti-LC3 antibody (anti–Microtubule associated proteins 1A/1B light chain 3; 1∶200, Novus Biologicals Inc., Littleton, CO), ii) anti-LAMP1 mouse monoclonal antibody LY1C6 (Anti-Lysosome-Associated membrane protein-1; 1∶200, Calbiochem, San Diego, CA), iii) rabbit poyclonal anti-Cleaved-Caspase-3 antibody (1∶400, Cell Signaling Technology Inc., Danvers, MA), iv) rabbit polyclonal anti-glial fibrillary acidic protein (GFAP) (1∶500; DakoCytomation, Denmark).

The antibodies were diluted in PBS containing 10% normal donkey serum (NDS) and 0.3% Triton X-100. After rinsing, primary antibodies were detected by incubating sections for one hour at room temperature in 1∶100 Cy2-conjugated donkey anti-rabbit IgG (H+L) or 1∶200 Cy3-conjugated donkey anti-mouse IgG (H+L) (Jackson Immuno Research Laboratories, West Grove, PA). Sections were counterstained with bisbenzimide (Hoechst # 33258, Sigma, St.Louis, MO), rinsed, coverslipped in 1∶1 PB-glycerol, and observed with a Nikon Eclipse E800 epifluorescence microscope under appropriate filters and a Leica TCS SP5 confocal laser scanning microscope (Leica, Mannheim, Germany).

Control sections to verify the specificity of the secondary antibodies was reacted similarly, except the primary antibody was omitted in incubation. No immunolabeling was seen in control sections (data not shown).

### TUNEL staining

Cell apoptosis was assessed by The DeadEnd™ Fluorometric TUNEL System (Promega Madison, WI, USA) following manufacturer's instructions. 4′,6-Diamidino-2-phenylindole (DAPI) (d9564) from Sigma-Aldrich was employed to stain nuclei.

### Double-Immuno fluorescence analysis of LC3 and TUNEL

Double-immuno fluorescence studies were performed for LC3 and TUNEL. The sections were incubated with LC3 primary antibody (L8918) from Sigma-Aldrich as described previously, then DeadEnd™ Fluorometric TUNEL System (Promega Madison, WI, USA) from Sigma-Aldrich was applied. 4′,6-Diamidino-2-phenylindole (DAPI) (d9564) was employed to stain nuclei.

### Immunoblotting

Animals were sacrificed 24 h after the increase in IOP, retinas were dissected from the sclera and then immediately homogenized in a glass-Teflon Potter homogenizer in an ice-cold lysis buffer containing 20 mM Hepes, pH 7.5/10 mM KCl/1.5 mM MgCl2/1 mM ethylenediaminetetraacetic acid (EDTA)/1 mM ethylene glycol tetraacetic acid (EGTA)/1 mM DTT/0.5% CHAPS/complete protease inhibitors; Roche Cat. No. 11 697 498 001) the homogenates were centrifuged at 12000 rpm for 15 min at 4°C. Protein concentration was determined using a Bradford assay #23236. Proteins extracts (50 µg) were separated on SDS-PAGE (12% polyacrilamide) and transferred to polyvinylidene difluoride membranes. Then the membranes were blocked in 5% nonfat milk in tris buffered saline (TBS)-T (200 mM Tris and 1.5 M NaCl with 0.1% Tween 20) and were incubated with primary antibody diluted in TBS-T overnight at 4°C.

The membranes were washed and incubated with secondary horseradish peroxidase-coupled antibodies (Santa Cruz Biotechnology, Santa Cruz, CA) in TBS-T for 1 hour at room temperature. After the final washes, the proteins were detected by enhanced chemiluminescence. The bands were quantified using Quantity One® 1-D Analysis Software (Bio-Rad Laboratories) and values were normalized with respect to tubulin. The values were then expressed as a percentage relative to the sham level of OD. The antibodies used were as follows: anti-LC3 (L8918) from Sigma-Aldrich (1∶1000), anti-b III tubulin (1637) from Millipore (1∶400), goat anti-mouse IgG-HRP (sc-2005) from Santa Cruz Biotechnology (1∶10000) and goat anti-rabbit IgG-HRP (sc-2004) from Santa Cruz Biotechnology (1∶10000).

### Labeling endocytosis *in vivo*

Endocytosis was documented by using one of two tracers: 10% horseradish peroxidase (HRP, Sigma-Aldrich, type VI-A) in saline, or 25 mg/ml 4.4 kDa fluorescein isothiocyanate (FITC)-labelled dextran (Sigma-Aldrich, St. Louis, MO) in saline. Each tracer was injected into the I/R eye and the control eye of two rats after the IOP was elevated; after three hours, the animals were killed, perfused, and retinas were collected and sectioned, as above, for histology. The two tracers were selected for the difference in their sizes: HRP (44 kDa) is a much larger molecule than dextran (4.4 kDa). To reveal HRP, sections were incubated with 10X 3,3 diaminobendizine/metal concentrate diluted to 1X in peroxide buffer (Roche Diagnostics GmbH, Mannheim, Germany) for 5 minutes, rinsed in dH_2_O for 5 minutes, counterstained with methyl green to label cell nuclei and coverslipped in PBS-glycerol (1∶1).Sections from animals that had received injections of FITC-labelled dextran were analyzed on a Nikon Eclipse E800 epifluorescence microscope, with the use of appropriate filters to document FITC-positive vacuoles; bisbenzimide (Hoechst # 33258, Sigma, St.Louis, MO) was used to counterstain cell nuclei.

### Cell counts

Nuclear profiles were counted under a 40x objective in five non-adjacent 100 µm-spaced sections from each retina all along the ganglion cell layer (GCL), starting at a distance 100 µm from the optic nerve head to the ora serrata. Nuclear profile counts (number of cells/500 µm) are expressed as mean among the animals±standard deviation (SD). The raw number of neurons in the GCL was corrected using the Abercrombie's correction factor [77], [78]. The statistical significance of the results was determined by hieralchical ANOVA followed by planned pair-wise comparisons with the Tukey test. P-values <0.05 are considered significant.

### 3-MA administration

3-Methyladeninde (Sigma-Aldrich, St. Louis, MO) was diluited in saline (0.9% NaCl) to a stock solution of 100 mM and vitreal injection was performed in the I/R retina (n = 3) with 3 µl (≈33 mM). Rats were injected at the end of I/R injury, under anaesthesia.
